# Multicistronic lentiviral vectors containing the FMDV 2A cleavage factor demonstrate robust expression of encoded genes at limiting MOI

**DOI:** 10.1186/1743-422X-3-14

**Published:** 2006-03-15

**Authors:** Dhanalakshmi Chinnasamy, Michael D Milsom, James Shaffer, James Neuenfeldt, Aimen F Shaaban, Geoffrey P Margison, Leslie J Fairbairn, Nachimuthu Chinnasamy

**Affiliations:** 1 Vince Lombardi Gene Therapy Laboratory, Immunotherapy Program, Aurora St. Luke's Medical Center, 2900 West Oklahoma Avenue, Milwaukee, WI 53215, USA; 2 Cancer Research UK Gene Therapy Group, Paterson Institute for Cancer Research, Christie Hospital NHS Trust, Wilmslow Road, Manchester, M20 4BX, UK; 3 Surgery Department, University of Wisconsin, Madison, WI 53792, USA; 4 Cancer Research UK Carcinogenesis Group, Paterson Institute for Cancer Research, Christie Hospital NHS Trust, Wilmslow Road, Manchester, M20 4BX, UK; 5 Division of Experimental Hematology, Cincinnati Children's Hospital Medical Center, Cincinnati, OH 45229, USA

## Abstract

**Background:**

A number of gene therapy applications would benefit from vectors capable of expressing multiple genes. In this study we explored the feasibility and efficiency of expressing two or three transgenes in HIV-1 based lentiviral vector. Bicistronic and tricistronic self-inactivating lentiviral vectors were constructed employing the internal ribosomal entry site (IRES) sequence of encephalomyocarditis virus (EMCV) and/or foot-and-mouth disease virus (FMDV) cleavage factor 2A. We employed enhanced green fluorescent protein (eGFP), *O*^6^-methylguanine-DNA-methyltransferase (MGMT), and homeobox transcription factor HOXB4 as model genes and their expression was detected by appropriate methods including fluorescence microscopy, flow cytometry, immunocytochemistry, biochemical assay, and western blotting.

**Results:**

All the multigene vectors produced high titer virus and were able to simultaneously express two or three transgenes in transduced cells. However, the level of expression of individual transgenes varied depending on: the transgene itself; its position within the construct; the total number of transgenes expressed; the strategy used for multigene expression and the average copy number of pro-viral insertions. Notably, at limiting MOI, the expression of eGFP in a bicistronic vector based on 2A was ~4 times greater than that of an IRES based vector.

**Conclusion:**

The small and efficient 2A sequence can be used alone or in combination with an IRES for the construction of multicistronic lentiviral vectors which can express encoded transgenes at functionally relevant levels in cells containing an average of one pro-viral insert.

## Background

Lentiviral vectors are efficient tools for gene transfer into various dividing and non-dividing target cells. They offer several advantages over other vectors, including stable integration into the host cell genome, lack of transfer of viral genes, and a relatively large capacity for therapeutic genes. A number of studies have demonstrated the ability of lentiviral vectors to achieve efficient and sustained transgene expression [1-6] and they have recently been approved for human clinical studies [7]. The majority of preclinical studies undertaken thus far have been conducted with the aim of transferring one therapeutic gene into target cells. However, many potential gene transfer applications require vectors that express more than one protein. These may include a therapeutic gene plus a selectable marker gene, multiple genes encoding different subunits of a complex protein or multiple independent genes that cooperate functionally. A number of strategies are employed in viral vectors to express multiple genes, including mRNA splicing, internal promoters, internal ribosomal entry sites, fusion proteins, and cleavage factors. The most commonly used strategy in the construction of two gene vectors is the insertion of an internal ribosome entry site (IRES) element between the two transgenes [8]. The IRES of encephalomyocarditis virus (EMCV) has been widely used to link two genes transcribed from a single promoter within recombinant viral vectors. However, there are a number of limitations using IRES elements, including their size and variability in expression of transgenes. In many cases it has been reported that a gene transcribed upstream of an IRES is expressed strongly whereas a gene placed downstream is expressed at lower levels [9,10].

Positive strand RNA viruses generally encode polyproteins that are cleaved by viral or host proteinases to produce mature proteins. Among other mechanisms many of these viruses are also known to contain 2A or similar peptide coding sequences to mediate protein cleavage. Foot and mouth disease virus (FMDV) is a picornavirus with an RNA genome that encodes a single poly-protein of approximately 225 kDa. This polyprotein is cleaved in the host cell to produce different protein products. A self-processing activity in FMDV leads to 'cleavage' between the terminal glycine of the 2A product and the initial proline of 2B. The exact mechanism of 2A/2B cleavage is not known. However, it has been hypothesized that the 2A sequence somehow impairs normal peptide bond formation between 2A glycine and 2B proline through a ribosomal skip mechanism without affecting the translation of 2B. The self-processing activity is conferred on heterologous fusion proteins by ~20 amino acids from the 2A region. The cleavage of the polyprotein product occurs at the C-terminal end of the 2A coding region, leaving this peptide fused to the upstream protein and releasing the downstream protein intact (with the addition of an N-terminal Proline).

Previously the FMDV 2A sequence has been successfully incorporated in to adeno-associated [11] and retroviral [12,13] vectors to construct multigene vectors. Multigene lentiviral vectors have been developed by other groups using strategies involving inclusion of IRES [14], multiple internal promoters [15,16] and differential splicing moieties [17]. More recently dual-gene lentiviral vectors were developed with synthetic bidirectional promoters [18].

Since the advent of the serious adverse effects observed in a clinical study of retroviral gene therapy for the treatment of X-linked SCID, it has become apparent that limiting MOI is desirable in order to minimize the risk of insertional mutagenesis [19-21]. Therefore, in order to determine whether the use of multi-cistronic vectors is realistically feasible for gene therapy applications, and to determine the most suitable co-expression strategy, it is essential to compare the performance of different vectors at limiting dilution. Herein we describe the development of HIV-1 based multigene lentiviral vectors using combinations of the FMDV 2A cleavage factor and the EMCV IRES. Bicistronic and tricistronic lentiviral vectors were able to coexpress 2 or 3 different proteins, albeit at levels that depend on the transgene and its location.

## Results

### Construction of multigene lentiviral vectors

**Figure 1:**
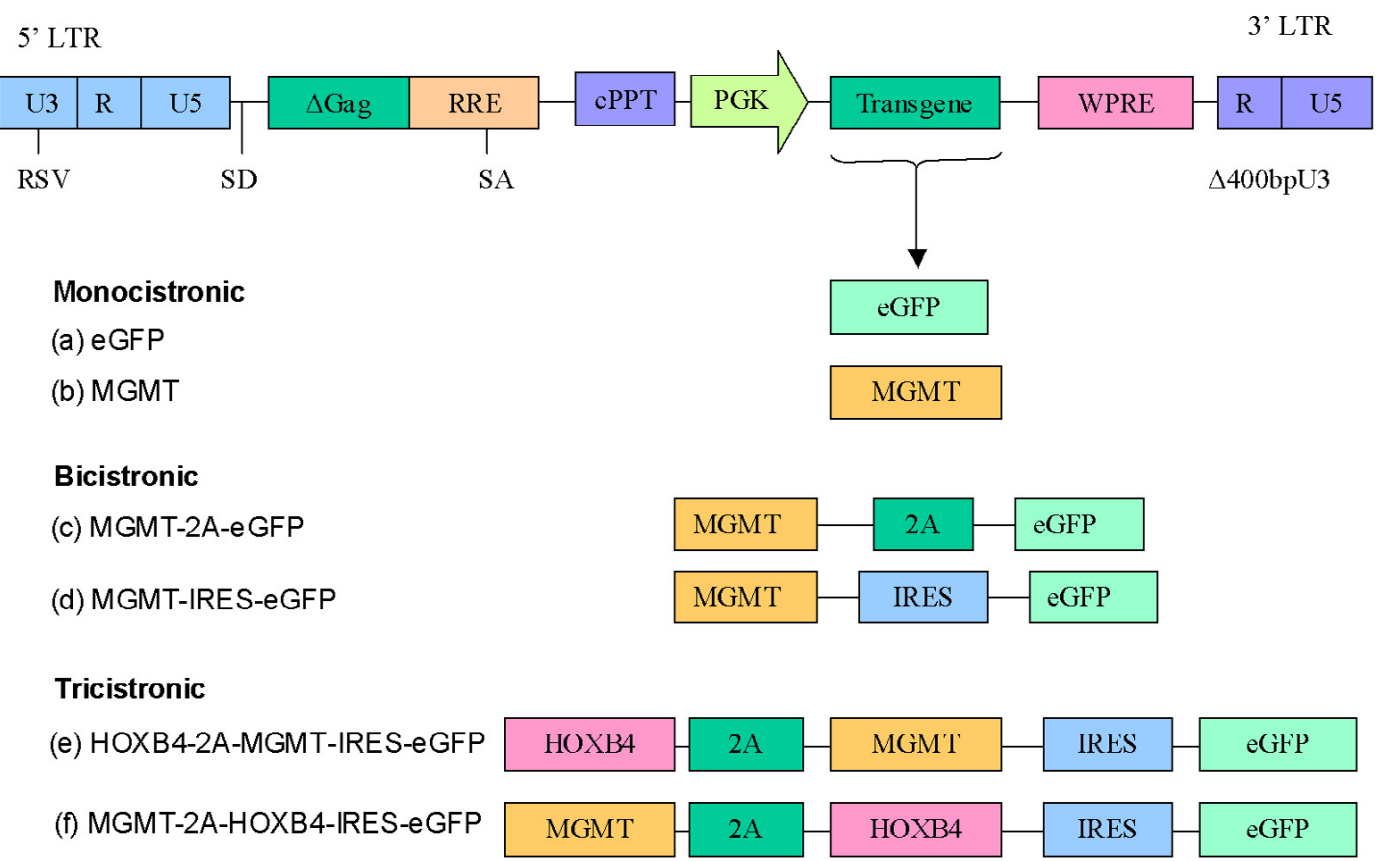
*Schematic diagram of HIV-1 based lentiviral vectors. Monocistronic vectors: *(a) eGFP, (b) MGMT, *Bicistronic vectors: *(c) MGMT-2A-eGFP, (d) MGMT-IRES-eGFP, *Tricistronic vectors: *(e) HOXB4-2A-MGMT-IRES-eGFP, (f) MGMT-2A-HOXB4-IRES-eGFP. The expression of the cassette is under the control of the human PGK promoter. The central polypurine tract (cPPT) is located upstream from the transgene and the posttranscriptional regulatory element of woodchuck hepatitis virus (WPRE) is placed downstream of the transgene. **RSV**, Rous sarcoma virus; **SD**, splice donor, **SA**, splice acceptor; ***Gag***, deleted *gag *region; **RRE**, Rev-responsive element; **LTR**, long terminal repeat; **IRES**, internal ribosome entry site sequence from encephalomyocarditis virus (EMCV); **2A**, sequence from foot-and-mouth disease virus; **eGFP**, enhanced green fluorescent protein; **MGMT**, *O*^6^-methylguanine DNA methyltransferase (proline 140 lysine mutant); **HOXB4**, homeobox transcription factor.

Multigene lentiviral vectors were constructed based on the previously described [22] self-inactivating (SIN) lentiviral vector backbone with central polypurine tract (cPPT) and woodchuck hepatitis virus post-transcriptional regulatory element (WPRE) in which transgene(s) are expressed under the control of the human PGK promoter. We constructed bicistronic and tricistronic vectors with the aid of IRES and 2A sequences (Figure 1). The cDNAs encoding eGFP, MGMT, and HOXB4 were used as model genes. Two types of bicistronic vectors were designed for the expression of two genes. In the first, we used the more common strategy of placing an IRES sequence in between the two cDNAs (MGMT and eGFP). In the second strategy we used FMDV 2A sequence to connect the two cDNAs. In this strategy MGMT (with its stop codon removed) was fused in frame with 2A and eGFP. Following translation, MGMT incorporates an extra 23 amino-acid peptide fused to its C-terminus whilst eGFP has an additional 7 amino-acid peptide fused to its N-terminus. A similar strategy was used to construct tricistronic vectors, with similar consequences for gene products downstream and upstream of the 2A cleavage site (with the exception of MGMT^P140K^-2A-HOXB4-IRES-eGFP, where HOXB4 has only a 4 amino-acid addition to its N-terminus). In tricistronic vectors, eGFP was always expressed as the last gene via the EMCV IRES.

### Vector production

Viral stocks were produced by co-transfecting each of the multigene transfer vector plasmids with packaging plasmid pCMVΔR8.91 and a plasmid encoding the vesicular stomatitis virus glycoprotein G (pMD.G) into 293T cells as described below. Viral particle containing supernatants were concentrated by centrifugation and titers were estimated by measuring HIV-1 *gag *protein p24 by ELISA. Estimated titers of bicistronic MGMT-IRES-eGFP (385 ± 270 ng/ml) and MGMT-2A-eGFP (368 ± 92 ng/ml); and tricistronic vectors HOXB4-2A-MGMT-IRES-eGFP (238 ± 126 ng/ml), and MGMT-2A-HOXB4-IRES-eGFP (380 ± 91 ng/ml) were comparable to those of monocistronic vectors encoding eGFP (383 ± 261 ng/ml) or MGMT (243 ± 92 ng/ml) alone (Table 1). The viral titers are shown as mean ± standard deviation from four independent experiments.

**Table 1:** Relative vector titers as measured by HIV-1 p24 *gag *protein.

Construct	ng p24/ml
a. eGFP	383 ± 261
b. MGMT	243 ± 92
c. MGMT-2A-eGFP	385 ± 270
d. MGMT-IRES-eGFP	368 ± 92
e. HOXB4-2A-MGMT-IRES-eGFP	238 ± 126
f. MGMT-2A-HOXB4-IRES-eGFP	380 ± 91

We transiently transfected each one of the above mentioned transfer vector plasmids with pCMVΔR8.91 and pMD.G into 293T cells as described in methods. Vector supernatants were harvested 72 h post transfection and concentrated by ultracentrifugation. HIV-1 *gag *protein (p24) was estimated in the concentrated vector preparations by ELISA. Values are expressed as nanograms of p24 (mean ± SD) from 4 independent experiments. Statistical analysis was performed using analysis of variance and Tukey's studentized range test. No statistically significant difference in titer (p24) was observed between the vectors tested.

### Bicistronic vectors

**Figure 2:**
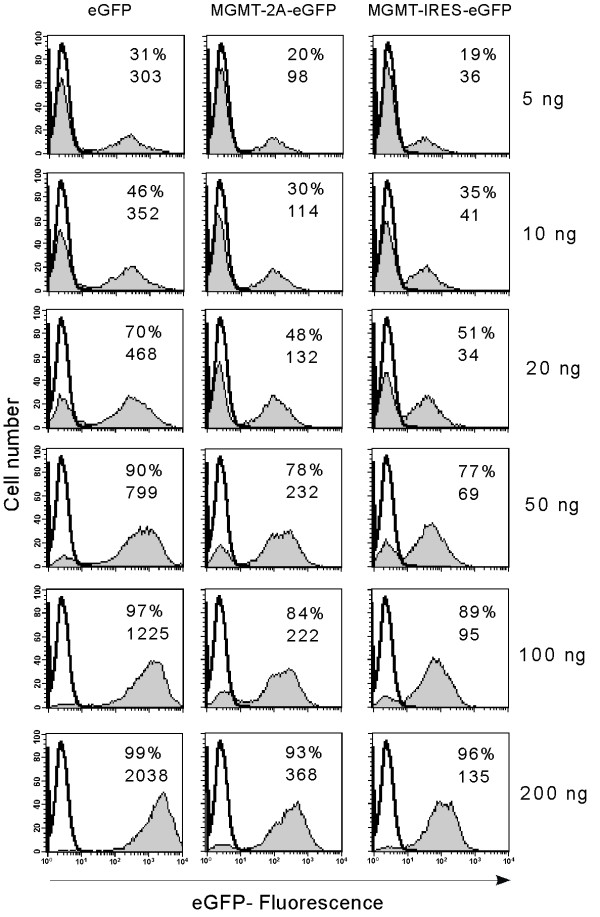
*Comparison of 2A and IRES-mediated eGFP (second gene) expression in bicistronic vectors*. To compare the level of second gene product (eGFP) expressed from either eGFP, MGMT-2A-eGFP or MGMT-IRES-eGFP vector, we transduced K562 cells with lentiviral vectors expressing eGFP downstream of either 2A or IRES sequences as shown Figure 1. All other sequences in the vectors were identical. K562 cells (5 × 10^4^) were transduced once with viral particles in the range of 5, 10, 20, 50, 100 and 200 ng of p24. Seven days after the transduction, cells were analyzed by flow cytometry for expression of eGFP. Untransduced K562 cells were used as control. Percentage of positive cells (given as % values on histograms) and the mean fluorescence intensity (given as numbers on histograms) were calculated using Cell Quest software.

We first compared the expression of MGMT and eGFP in cells transduced with bicistronic vectors with that seen in cells transduced with monocistronic vectors expressing either MGMT or eGFP alone. To do this, we transduced Hela and K562 cells with increasing MOI as judged by the p24 estimations. The relative levels of reporter gene expression seen post transduction may be a reflection of a number of variables including transduction efficiency, number of copies of integrated transgene, and efficiencies of transcription and translation. We therefore firstly examined the performance of IRES or 2A based bicistronic vectors in terms of their relative expression of the second gene, eGFP, by measuring the mean fluorescence intensity by flow cytometry. Figure 2 shows the mean fluorescence intensity and percentage of eGFP positive cells of a representative example of K562 cells transduced with increasing MOI. Cells were analyzed 7 days after a single round of transduction. Untransduced cells were used as controls for comparison.

**Figure 3:**
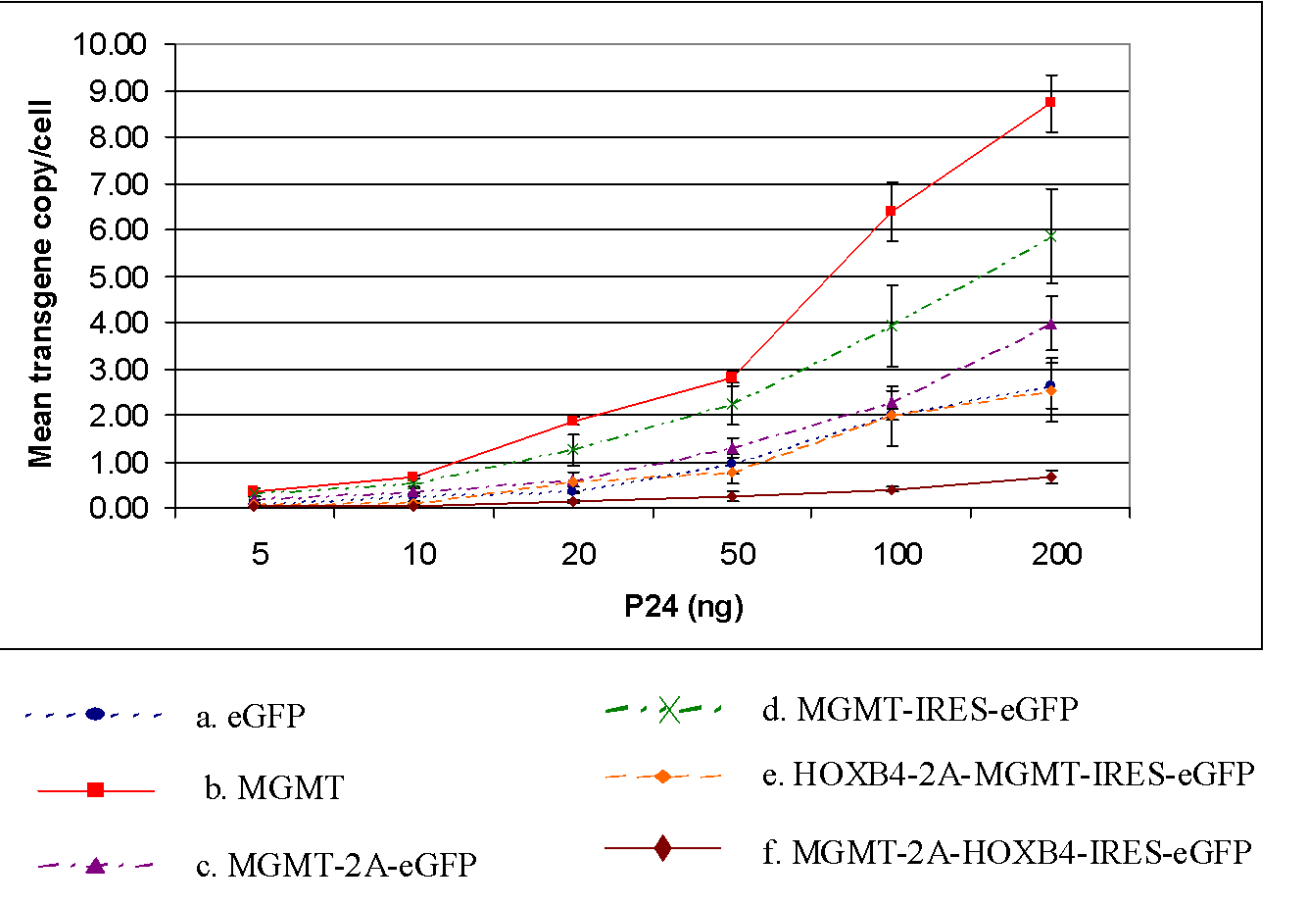
*Analysis of average transgene copy number by real-time quantitative PCR*. To compare the average transgene copies among the transduced K562 cells real time Q-PCR analysis was carried out using primers specific for sequences located within WPRE region of the vector.(a) eGFP, (b) MGMT, (c) MGMT-2A-eGFP, (d) MGMT-IRES-eGFP, (e) HOXB4-2A-MGMT-IRES-eGFP, (f) MGMT-2A-HOXB4-IRES-eGFP. Values are expressed as mean ± SEM of 4 to 6 independent observations.

**Figure 4:**
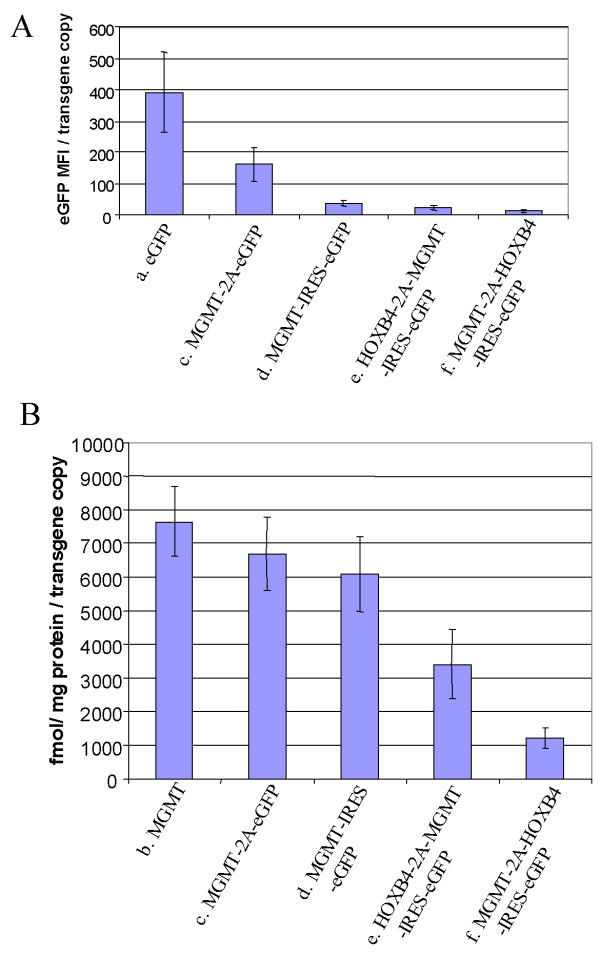
(A) *EGFP expression (MFI) in K562 cells calculated per copy number from the flow cytometry data*. Samples were selected among the cells having close to an average copy number of ~1. (a) eGFP, (c) MGMT-2A-eGFP, (d) MGMT-IRES-eGFP, (e) HOXB4-2A-MGMT-IRES-eGFP, (f) MGMT-2A-HOXB4-IRES-eGFP. (B). *MGMT expression measured as biochemical activity in K562 cells following lentiviral transduction*. Activity is presented as fmol/mg protein/transgene copy number. (b) MGMT, (c) MGMT-2A-eGFP, (d) MGMT-IRES-eGFP, (e) HOXB4-2A-MGMT-IRES-eGFP, (f) MGMT-2A-HOXB4-IRES-eGFP. All the values are expressed as mean ± SEM of 4 to 6 independent observations.

Following transduction with relatively low MOIs, (5–50 ng p24), we noticed a slightly lower efficiency of transduction by the two bicistronic vectors compared to the monocistronic eGFP vector as assessed by the percentage of eGFP positive cells (Figure 2). At higher MOI, however, this appeared to normalize, with comparable levels of transduction by all three vectors. In contrast, when mean vector copy number was assessed by Q-PCR, MGMT-IRES-eGFP vector transduced cells had more integrated copies at any given MOI than MGMT-2A-eGFP or eGFP transduced cells (Figure 3). Flow cytometric analysis of eGFP fluorescence is a convenient quantitative measurement of expression levels of this marker gene in transduced cell populations. To compare more directly the levels of eGFP expression between vectors, we normalized MFI to proviral copy number. Figure 4A shows the eGFP-expression from the various transduced populations expressed as MFI per copy number and Table 2 shows these data relative to expression from the monocistronic construct. It is clear that both bicistronic vectors express eGFP with lower efficiency than the monocistronic one. However, whilst MGMT-2A transduced K562 cells exhibited around 2.5-fold lower relative eGFP expression than eGFP-transduced K562 cells, expression from the IRES vector was much worse (around 10-fold lower than from the monocistronic vector and 4-fold worse than the 2A bicistronic vector).

**Table 2:** Relative expression of MGMT and eGFP

Vector	Relative MGMT Expression	Relative eGFP Expression
MGMT	1.00 ± 0.14	NA
eGFP	NA	1.00 ± 0.33
MGMT-2A-eGFP	0.87 ± 0.14	0.41 ± 0.14
MGMT-IRES-eGFP	0.80 ± 0.15	0.10 ± 0.02
HOXB4-2A-MGMT-IRES-eGFP	0.45 ± 0.13	0.06 ± 0.02
MGMT-2A-HOXB4-IRES-eGFP	0.16 ± 0.04	0.04 ± 0.01

NA-Not applicable

Column 1: MGMT activity per average copy number is calculated relative to the expression in monocistronic vector. Both bicistronic vectors are equally good for MGMT expression (as opposed to eGFP expression). Tricistronic vectors are less efficient than mono and bicistronic vectors for MGMT expression, but downstream sequences in the tricistronic vectors also affect MGMT expression.

Column 2: EGFP expression per copy number expressed relative to monocistronic eGFP vector. From this it can be seen that bicistronic 2A vector is much better than IRES based vector for eGFP expression. EGFP consistently expressed poorly when placed downstream of IRES in either bi or tricistronic vectors. All the values are expressed as mean ± SEM of 4 to 6 independent observations.

When expression of MGMT activity was determined per proviral copy, it was also clear that the bicistronic vectors showed closely similar levels of expression to each other and to that of the monocistronic MGMT vector (Figure 4B and Table 2). Expression of MGMT-2A-eGFP cassette produces MGMT protein with an extra 23 amino acid peptide fused to C-terminus. The presence of this extra 23 amino acid peptide did not seem to interfere with the activity of MGMT since levels from the IRES vector were comparable (Figure 4B and Table 2).

**Figure 5:**
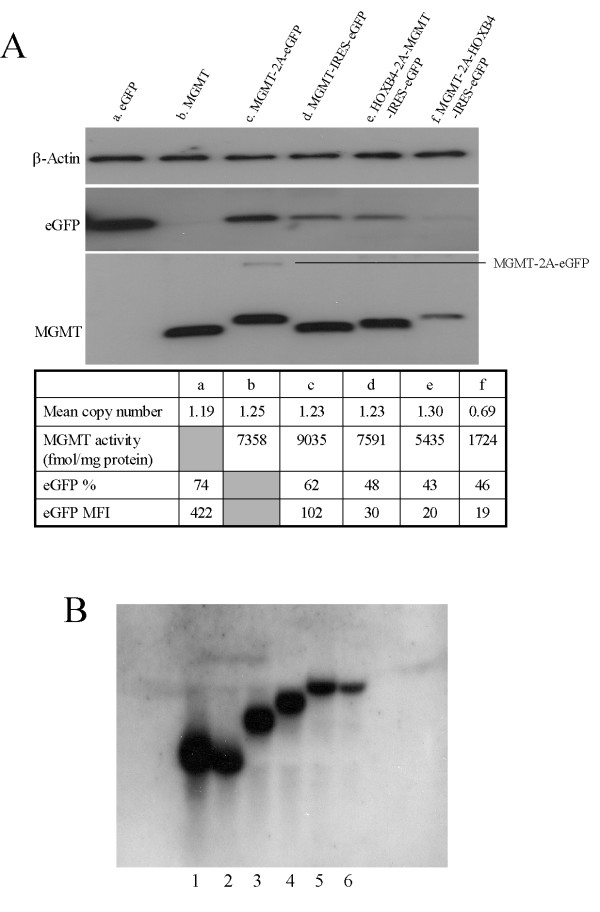
(A). *Western blot analysis of β-actin, eGFP and MGMT expression in transduced K562 cells*. Lanes a. eGFP, b. MGMT, c. MGMT-2A-eGFP, d. MGMT-IRES-eGFP, e. HOXB4-2A-MGMT-IRES-eGFP, f. MGMT-2A-HOXB4-IRES-eGFP. Mean copy number, MGMT activity, percentage of eGFP positive cells and MFI of the given samples are indicated in the table. (B). *Northern blot analysis of vector-derived transcripts in transduced K562 cells*. Lanes 1. eGFP, 2. MGMT, 3. MGMT-2A-eGFP, 4. MGMT-IRES-eGFP, 5. HOXB4-2A-MGMT-IRES-eGFP, 6. MGMT-2A-HOXB4-IRES-eGFP.

Western blot analysis was carried out to detect the levels of MGMT protein. MGMT-2A-eGFP transduced cells produce MGMT protein with a 2A peptide attached to their C terminus, and this migrates differently from its wild-type counterpart. MGMT-2A-eGFP transduced cells also showed a very minor higher molecular weight band indicating the presence of uncleaved MGMT-2A-eGFP fusion protein (Figure 5A). We observed this minor fraction of uncleaved fusion protein only in MGMT-2A-eGFP transduced cells and not in other tricistronic vectors containing 2A. MGMT and MGMT-IRES-eGFP vector transduced cells showed a single band corresponding to the MGMT protein of the expected size (Figure 5A). Northern blot analysis of total RNA isolated from monocistronic and bicistronic virus transduced K562 cells showed vector-derived transcripts proportional to their MGMT and eGFP protein levels as detected by Western blot analysis (Figure 5A and 5B).

**Figure 6:**
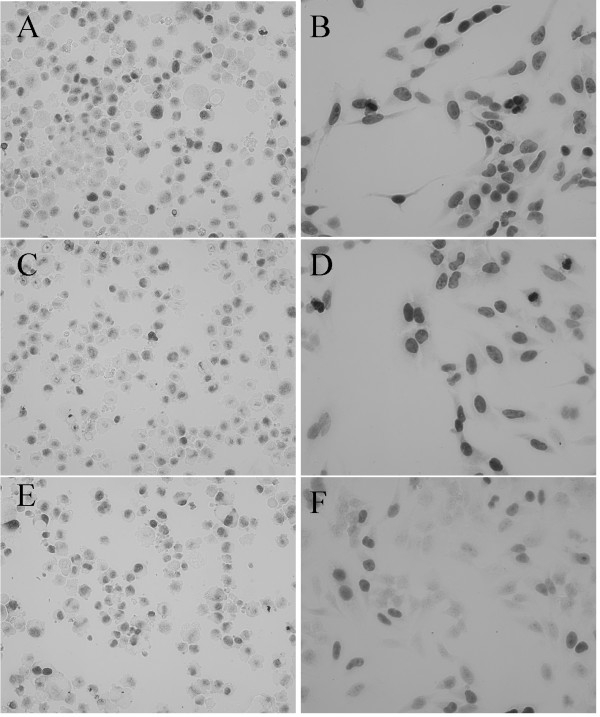
*Immunocytochemistry demonstrating MGMT expression in K562 and Hela cells*. Immunocytochemistry was performed with rabbit polyclonal anti-human MGMT antisera as described in methods. Immunocytochemical detection of MGMT shows clear nuclear localization. A, C, E are K562 cells transduced with MGMT-2A-eGFP, HOXB4-2A-MGMT-IRES-eGFP and MGMT-2A-HOXB4-IRES-eGFP respectively. B, D, F are Hela cells transduced with MGMT-2A-eGFP, HOXB4-2A-MGMT-IRES-eGFP and MGMT-2A-HOXB4-IRES-eGFP respectively.

Intracellular localization of MGMT protein to the nucleus was demonstrated by immunocytochemistry (ICC) (Figure 6). Untransduced control K562 cells were negative for MGMT expression as previously reported (data not shown) [22] whereas the transduced cells showed nuclear staining, which in many cases was intense, indicating that MGMT is localized to the nucleus as anticipated (Figure 6). Hence, the presence of the 23 extra amino acids did not appear to impair the nuclear localization of the MGMT protein in MGMT-2A-eGFP transduced K562 or Hela cells.

### Tricistronic vectors

Next we explored the possibility of directing the expression of three transgenes in a lentiviral vector by using a combination of 2A and IRES sequences. Tricistronic vectors were constructed with the aid of both IRES and 2A sequences connecting the three cDNAs (Figure 1). In these constructs the 2A sequence was used to connect the first two transgene, whilst the third gene was expressed *via *the IRES sequence. All the remaining components of the vector backbone were the same as those of monocistronic and bicistronic vectors. Two tricistronic lentiviral vectors were constructed as described in methods, HOXB4-2A-MGMT-IRES-eGFP and MGMT-2A-HOXB4-IRES-eGFP.

**Figure 7:**
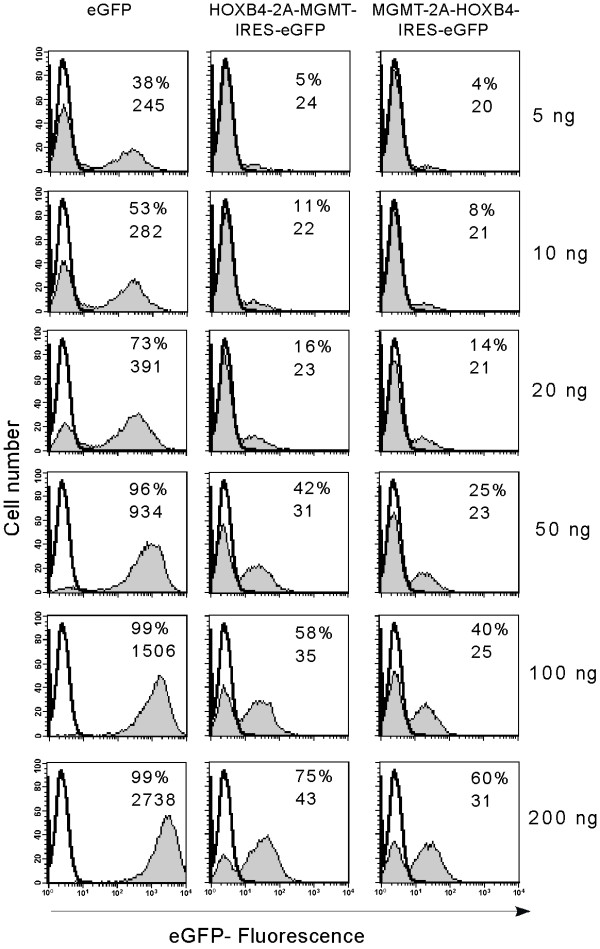
*Comparison of IRES-mediated third gene expression in tricistronic vectors*. To compare the levels of third gene product expressed downstream of IRES, we transduced K562 cells with tricistronic lentiviral vectors with eGFP placed as the third transgene as shown in Figure 1. K562 cells (5 × 10^4^) were transduced once with viral particles in the range of 5, 10, 20, 50, 100 and 200 ng of p24. Seven days after transduction, cells were analyzed by flow cytometry for expression of eGFP. Untransduced K562 cells were used as control. The percentage of eGFP positive cells and MFI are given in each histogram for eGFP, HOXB4-2A-MGMT-IRES-eGFP and MGMT-2A-HOXB4-IRES-eGFP transduced cells.

To determine the efficiency of coexpression of 3 genes, K562 and Hela cells were transduced at various MOI. First we examined the performance of tricistronic vectors for relative expression of the third gene eGFP by measuring the MFI by flow cytometry 7 days following a single round of transduction. Figure 7 shows the MFI and percentage of eGFP positive cells of a representative example of K562 cells transduced with increasing MOI. Untransduced cells were used as controls for comparison. There was a lower efficiency of transduction of the tricistronic vectors compared to that of monocistronic (eGFP) or bicistronic vectors as assessed by the percentage of eGFP positive cells following transduction (Figure 7). When copy number was assessed by Q-PCR, it was evident that the mean proviral copy per cell was less for tricistronic vectors at a given level of p24, than for monocistronic MGMT and bicistronic vectors with the exception of monocistronic eGFP vector. When eGFP levels (MFI) were normalized to proviral copy number it was again clear that IRES-mediated eGFP expression was much less efficient than that from monocistronic or 2A based bicistronic vectors. The level of eGFP per proviral copy was progressively lower in tricistronic vectors and MGMT-2A-HOXB4-IRES-eGFP construct expressed lowest level (Figure 4A, Table 2). We next analyzed MGMT expression as measured by the activity from each of the vectors. MGMT levels per proviral copy were reduced (2 to 6 fold) with tricistronic compared with monocistronic vectors, and also reduced (2 to 5 fold) compared to bicistronic vectors (Figure 4B, Table 2).

**Figure 8:**
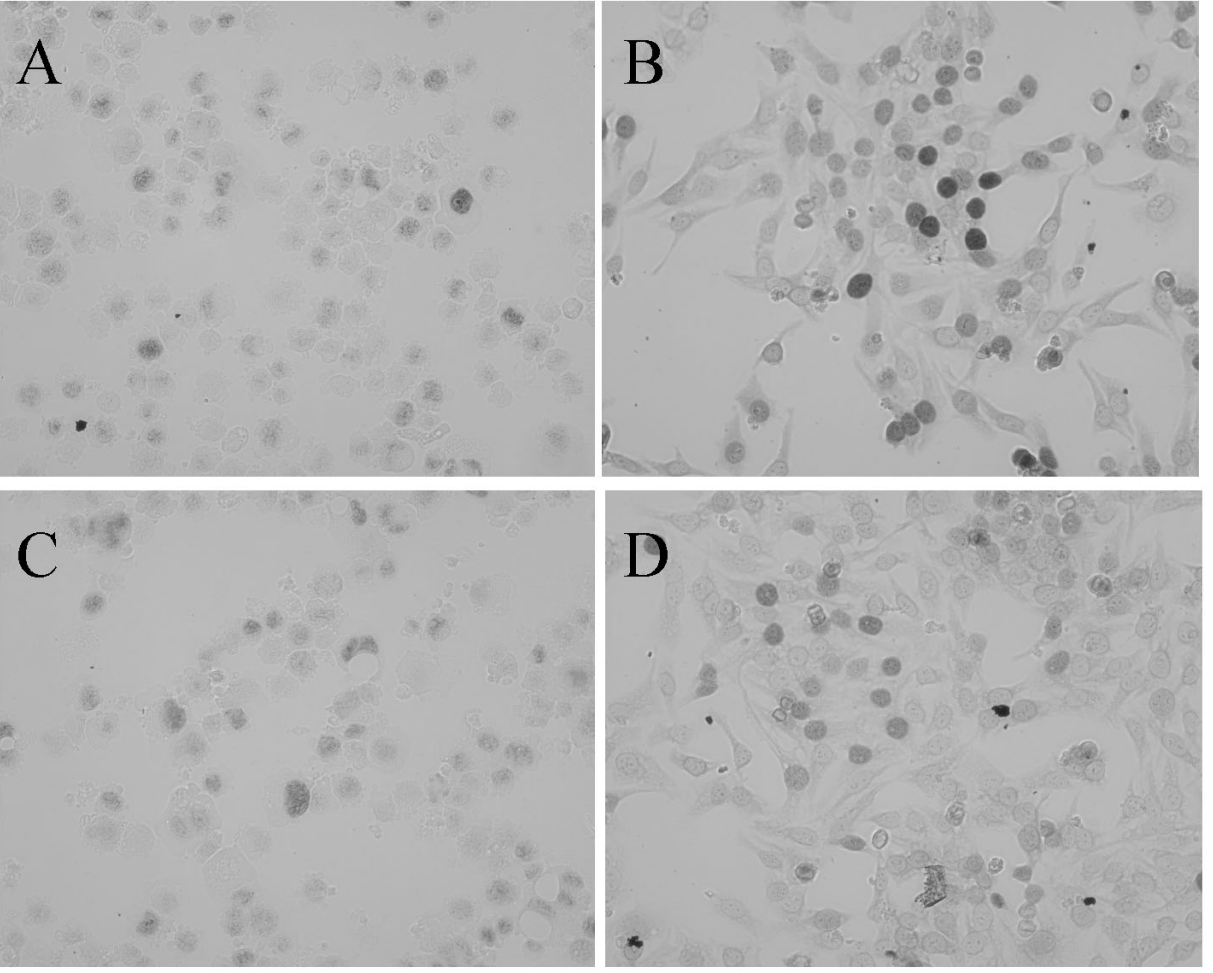
*Immunocytochemistry demonstrating expression of HOXB4 in transduced K562 and Hela cells*. Immunocytochemistry was performed with rat anti HOXB4 as described in methods. Immunocytochemical detection of HOXB4 shows clear nuclear localization. A and C are K562 cells transduced with HOXB4-2A-MGMT-IRES-eGFP and MGMT-2A-HOXB4-IRES-eGFP respectively. B and D are Hela cells transduced with HOXB4-2A-MGMT-IRES-eGFP and MGMT-2A-HOXB4-IRES-eGFP respectively.

To verify that the fusion proteins produced by the multigene cassettes were cleaved efficiently, MGMT protein expression was again assessed by western blot analysis using an antiserum directed against MGMT. Both HOXB4-2A-MGMT-IRES-eGFP and MGMT-2A-HOXB4-IRES-eGFP transduced cells produced a single band that was slightly larger than that produced from cells transduced with the MGMT monocistronic vector, owing to the addition of 2A peptide sequence (Figure 5A). Northern blot analysis of total RNA isolated from K562 cells transduced with HOXB4-2A-MGMT-IRES-eGFP and MGMT-2A-HOXB4-IRES-eGFP showed vector-derived transcripts expressed at a level proportional to their MGMT and eGFP protein levels as detected by Western Blot analysis (Figures 5A and 5B). Notably, the levels of RNA were proportionately lower in tricistronic vector transduced cells compared with bicistronic vector-transduced cells (Figure 5B). The correct subcellular localization of expressed MGMT and HOXB4 to the nucleus was demonstrated by ICC in transduced K562and Hela cells (Figures 6 and 8). Taken together, these data demonstrate the ability of tricistronic vectors to permit the simultaneous expression of three transgenes, albeit with substantial differences in both transduction and expression efficiencies. An aliquot of the transduced K562 cells were cultured over a period of 6 months revealed sustained transgene expression (data not shown). We also transduced primary mouse embryonic fibroblasts and OP9 bone marrow stromal cells with all the vectors described herein and noticed efficient expression of multiple genes similar to the human cells indicating that these vectors are functional in multiple cell types (data not shown).

## Discussion

Currently there are several types of gene delivery vectors available to deliver one or two genes into target cells. An increasing demand for more complex multicistronic vectors has arisen in recent years for various applications both in basic research and clinical gene therapy. Herein we described a new method to coexpress multiple transgenes efficiently in HIV-1 based lentiviral vectors. We constructed bicistronic and tricistronic lentiviral vectors using combinations of a self-processing 2A cleavage factor and IRES and undertook systematic analysis of the expression of selected marker genes. In this report we describe bicistronic and tricistronic lentiviral vectors. These multigene vectors can successfully co-express 2 or 3 transgenes under the direction of a single promoter. All the vectors described in this study produced high titer vector stocks comparable to the monocistronic vectors. They were also able to transduce multiple target cells of human and murine origin efficiently. However, there were differences in the level of transgene expression among the vectors depending on the size, position and total number of transgenes placed within the expression cassette; and type of transgene involved. Bicistronic vectors based on the 2A cleavage factor were more efficient in the co-expression of two transgenes than IRES based vectors. Indeed, co-expression mediated by the 2A motif was superior to internal ribosome entry across a range of different vector MOIs, and it is of import that this differential was maintained at a limiting copy number. Thus, 2A represents an attractive alternative to currently used systems for the co-expression of two proteins in lentiviral vectors.

A major advantage of using the 2A cleavage factor in the construction of multicistronic vectors is its small size compared to internal promoters or IRES sequences. Given the packaging constraints on lentiviral vectors, minimizing the size of sequences required to enable co-expression is important in maximizing the capacity for therapeutic sequences. In addition, efficient co-expression of both genes is ensured as we have shown in the case of MGMT-2A-eGFP. The 2A sequence efficiently promoted the generation of predicted cleavage products from the artificial fusion protein in transduced cells. Previous studies with oncoretroviral [13,23,24] and AAV [11] vectors have shown the feasibility of using the 2A sequence for the expression of multiple transgenes. Incomplete cleavage of 2A mediated fusion products has previously been reported in AAV [11] and retroviral vectors [12,25]. In our hands, the efficiency of cleavage was construct dependent, with the MGMT-2A-eGFP cassette leading to some (approximately 6–8%) uncleaved product, whilst those cassettes incorporating HOXB4 showed apparent 100% cleavage. Although the reason for incomplete cleavage remain obscure, it is not unreasonable to speculate that differences in fusion protein secondary structure might influence this.

In addition to efficient generation of cleavage products, it is important that these are transported to the appropriate compartment of the cell where their action is required. As shown by the nuclear localization of HOXB4 and MGMT in our study, the addition of 2A sequences did not adversely affect the trafficking of these two proteins. Recently Szymczak et al [24] reported the construction of a multicistronic retroviral vector using multiple 2A cleavage factors or similar sequences with efficient coexpression of complete T cell receptor complex proteins. They showed that a 2A like peptide linked retroviral vector could be used to express all of the four CD3 proteins (CD3ε,γ,δ,ζ), appropriately localized to the membrane and that this restored T cell development in CD3 deficient mice. However in another recent report, mistargetting of second gene products was observed dependent on the context in which they were expressed [26]. It will be important; therefore, to empirically test any co-expression cassette to ensure that localization of transgene products is appropriate. Szymczak et al used four separate 2A sequences from different viruses, which share a conserved sequence. To avoid recombination they changed codon usage by introducing silent mutations within 2A sequences. A similar approach in lentiviral vectors might allow efficient delivery of multiple genes linked with multiple 2A cleavage factors without the need to use IRES sequences. However, whether or not recombination would be a problem if identical sequences were used, may be worth establishing.

One particular attraction of this 2A-based strategy is in applications in which it is desirable to coexpress two or more therapeutic genes in comparable amounts as in the case of two subunits of a functional protein (e.g. enzyme, cytokines). Previously described lentiviral vectors based on IRES or multiple internal promoters [16] have revealed inconsistent levels of expression of individual transgenes within the expression cassette. From our data summarized in Table 2, it is clear that the relative levels of MGMT and eGFP expression from the bicistronic 2A-based vector were higher than IRES based vector. In contrast, expression of eGFP from the IRES-containing vector was around one fifth that of MGMT. Although this is an improvement on other reports of IRES-containing lentiviral vectors [16], such a discrepancy in expression levels of the upstream and downstream genes would probably be detrimental to certain therapeutic applications. 2A based multigene vectors, thus offer the unique advantage of better coexpression of two or more desired transgenes. It is of particular interest that this comparison was made at limiting MOI using expression cassettes whose transcription was driven by a clinically relevant human cellular promoter. Hence we can conclude that a 2A mediated co-expression strategy is significantly improved over an approach using the EMCV IRES when lentiviral vectors are used to infect cells at a level which is appropriate to gene therapy applications, where a major concern may be minimizing the risk of insertional mutagenesis.

In addition to the potential for intracellular mislocalisation of protein, the addition of 2A peptide [[17] additional amino acids in this case) to the first gene product might also interfere with the function of a given protein, and again this will have to be determined empirically for each application. In our experience addition of the 2A peptide did not affect the function of MGMT protein as neither its DNA repair activity nor nuclear localization were altered. Moreover, recent studies indicated that HOXB4 expressed using the 2A strategy retains its ability to support hematopoietic reconstitution by murine hematopoietic stem cells [25,27]. A further issue might be immunogenicity due to the attachment of the 2A peptide-adduct to a therapeutic protein. Although these problems are not encountered in two recent murine *in vivo *studies [24,25], further studies in multiple species are needed to understand this issue. More recently Fang et al [28] successfully engineered a furin cleavage site next to the 2A sequence to eliminate any possible adverse effects that might be caused by having a 2A peptide residue on a therapeutic protein.

## Conclusion

In conclusion, we have developed multigene lentiviral vectors, incorporating 2A and IRES sequences that efficiently mediated the co-expression of two or three transgenes in multiple cell types. Multicistronic vectors are useful for various basic laboratory studies and gene therapy applications. They could be used in genetic immunotherapy strategies where more than one gene products are necessary to mount an effective immune response [29]. In chemoprotective strategies, expression of multiple drug resistance genes in hematopoietic stem cells would help to protect the hematopoietic compartment from a variety of cancer chemotherapeutic drugs [30]. These vectors may also be useful for the treatment of neurodegenerative diseases such as Parkinson's disease where up to 3 or 4 genes may be required for the effective production and transportation of dopamine [31].

## Methods

### Plasmid construction

The lentiviral vectors used in this study are in pRRL.PPT.PGK.X.W.SIN backbone and pRRL.PPT.PGK.eGFP.W.SIN and pRRL.PPT.PGK.MGMT^P140K^.W.SIN are described previously [22]. Multigene cassettes were constructed in pSF91m3 vectors and flanked by a 5' Not I site and 3' BamH I site, complete details of construction steps are given in the following paragraph. A sub cloning step was required, in which each pSF91m3 plasmid was digested with the aforementioned restriction enzymes having one or both ends of the expression cassettes filled-in and transferred into pBluescript KS+ or pGEM7zf(-) (Promega, Madison, WI) before final transfer into lentiviral vector pRRL.PPT.PGK.eGFP.W.SIN replacing eGFP at the BamH I and Sal I sites.

The expression cassettes: MGMT^P140K^-2A-eGFP and HOXB4-2A-MGMT^P140K^-IRES-eGFP and have been previously described [25]. In brief: **(i) HOXB4-2A-MGMT^P140K^-IRES-eGFP**. HOXB4 was amplified from its cDNA using the oligonucleotides TTGCGGCCGCCATGGCTATGAGTTCTTTTTTGATC and TTCTCGAGAGAGCGCGCGGGGGCCTC, following which it was digested with *Not *I and *Xho *I. FMDV 2A was amplified using the oligonucleotides TTCTCGAGTGAAACAGACTTTGAATTTTGACC and CCGGTGGATCCCATAGAATTCC, following which it was digested with *Xho *I and *Bam*H I. MGMT^P140K ^was amplified from its cDNA using the oligonucleotides GGTACCCGGAGATCTATGGACAAGG and TTGGATCCTCAGTTTCGGCCAGCAGG, following which it was digested with *Bgl *II and *Bam*H I. The EMCV IRES was amplified from pIRES2-eGFP (Clontech) using the oligonucleotides TACCGCGGGCCCGAGATCTGCCCCTCTC and CCGGATCCCATGGTTGTGGCCATATTATCA, followed by digest with *Bgl *II and *Bam*H I. eGFP was also amplified from pIRES2-eGFP using the primers GACTCTAGAAGATCTATGGTGAGC and TTGGATCCTTACTTGTACAGCTC, following which it was digested with *Bgl *II and *Bam*H I. The HOXB4-2A-MGMT^P140K^-IRES-eGFP cassette was then sequentially assembled as a *Not *I/*Bam*H I restriction fragment. **(ii) MGMT^P140K^-2A-HOXB4-IRES-eGFP. **MGMT was amplified from its cDNA using the oligonucleotides TTGCGGCCGCCATGGACAAGGATTGTGAAATG and TTCTCGAGAGTTTCGGCCAGCAGGC, following which it was digested with *Not *I and *Xho *I. FMDV 2A was amplified as described in (i), followed by digestion with *Xho *I and *Eco*R I. HOXB4 was amplified from its cDNA using the oligonucleotides TTGAATTCTATGGCTATGAGTTCTTTTTTGATC and TTGGATCCCTAGAGCGCGCGGGGGCCTC, followed by digest with *Eco*R I and *Bam*H I. Both the EMCV IRES and eGFP were isolated and digested as described in (i). The MGMT^P140K^-2A-HOXB4-IRES-eGFP cassette was then sequentially assembled as a *Not *I/*Bam*H I fragment. **(iii) MGMT^P140K^-2A-eGFP**. MGMT^P140K ^was amplified and digested as described in (ii). Both FMDV 2A and eGFP were isolated and digested as described in (i). The MGMT^P140K^-2A-eGFP cassette was then sequentially assembled as a *Not *I/*Bam*H I fragment. **(iv) MGMT^P140K^-IRES-eGFP. **MGMT^P140K^, EMCV IRES and eGFP were amplified and digested as described in (i). The MGMT^P140K^-IRES-eGFP cassette was then sequentially assembled as a *Bgl *II/*Bam*H I fragment.

### Cell culture

K562 and Hela cell lines were obtained from the American Type Culture Collection (ATCC, Manassas, VA). The human embryonic kidney cell line 293T and Hela cells were cultured at 37°C with 5% CO_2 _in Dulbecco Modified Eagle's Medium (Invitrogen, Carlsbad, CA) supplemented with 10% fetal bovine serum (FBS) (HyClone, CA). K562 cells were cultured in RPMI 1640 medium supplemented with 10% FBS and 2 mM glutamine.

### Virus production and titering

Replication-defective lentiviral vector particles were produced by 3-plasmid transient transfection of 293T cells as previously reported [32]. Briefly, 293T cells plated to ~70% confluency are cotransfected with pMD.G, pCMVΔR8.91, and the appropriate gene transfer vector plasmid by calcium phosphate transfection method. Viral particles were concentrated by centrifugation at 50,000×g for 90 minutes. The resulting pellets were resuspended in X-VIVO 10 medium (Cambrex Bio Science, Walkersville, MD) and stored at -80°C. Concentrated viral preparations were tested by ELISA for HIV-1 p24 (*gag*) antigen. The possibility of the generation of replication-competent lentivirus (RCL) was tested by checking for the presence of the viral protein p24 in the culture media of stably transduced 293T cells. All the samples tested were negative for RCL particles.

### Lentiviral transduction of K562 and Hela cells

K562 and Hela cells were transduced with lentiviral vectors at various multiplicity of infection (MOI) in the presence of 10 μg/ml protamine sulphate. Transduced cells were washed 48 hours after transduction and analyzed 7 days later. An aliquot of the transduced cells was cultured over a period of 6 months to study the long-term gene expression. Whole-cell population was used rather than selected clones in all of our experiments.

### Flow cytometric analysis

Fluorescence-activated cell sorter (FACS) analysis was carried out for the detection of cellular expression of eGFP using a FACScan flow cytometer (Beckton-Dickinson) with the FL1 detector channel. The data were acquired and analyzed with CellQuest software (BD). Untransduced cells were used as controls. Mean fluorescence intensity (MFI) was used as an indicator of relative expression of eGFP on given cells. Results were presented as a percent of positive cells and MFI.

### MGMT activity

Lentivirally transduced and untransduced control K562 cells were harvested 4 weeks following the transduction and the biochemical activity of MGMT in the cell extracts were determined by quantitation of the transfer of [^3^H]-methyl groups from [^3^H]-MNU-methylated calf thymus DNA substrate to MGMT protein as described previously [22,33]. MGMT activity was presented as femto moles of methyl group transferred per milligram of total protein. Protein concentrations in the cell extracts were determined by Bradford assay using bovine serum albumin (BSA) as standard.

### Western blotting

Western blot analysis was carried out to assess expression of B-actin, eGFP and MGMT proteins in transduced cells. Cell extracts containing 5 μg of protein were loaded onto polyacrylamide gel and separated. Proteins were transferred to PVDF membranes and blocked in 5% nonfat milk in TBS. The membrane was briefly washed with TBS/Tween and incubated with mouse monoclonal anti human β-actin (Sigma, St. Louis, MO), mouse monoclonal anti GFP (Clontech) or rabbit antihuman MGMT antisera over night at 4°C. The membrane was then washed and incubated with appropriate horseradish peroxidase conjugated secondary antibody for one hour at room temperature. The membrane was put through a final wash step and incubated with chemiluminescent substrate (Pierce, Rockford, IL) for five minutes at room temperature before being exposed to autoradiography film.

### Northern blotting

Total RNA was isolated from transduced K562 cells using RNeasy kit (Qiagen, Chatsworth, CA). RNA (10 μg per lane) samples were subjected to electrophoresis through a 1% denaturing formaldehyde agarose gel and transferred to a nylon membrane by capillary blotting. The blot was then hybridized with a WPRE specific probe, labeled with the Roche PCR DIG probe synthesis kit and Roche High Prime DNA labeling and detection kit (Roche Diagnostics GmbH, Mannheim, Germany) and the signal detected using Biomax Light Film.

### Immunocytochemistry

Immunocytochemistry was performed to detect the expression and intracellular localization of MGMT and HOXB4 proteins in transduced cells. Cytospin preparations of transduced K562 cells and Hela cells grown on the chamber slides were stained with rabbit anti human MGMT antiserum [34] or rat anti human HOXB4 antibody (University of Iowa, Clone I12). Biotin conjugated goat anti rabbit or goat anti rat IgG was used as a secondary antibody and then a horseradish peroxidase conjugated avidin-biotin system (Dako, Carpinteria, CA) was used to detect MGMT or HOXB4 with Diaminobenzidine (DAB) as chromogen. The slides were examined using a Nikon microscope, and the images were captured and analyzed using Image Pro plus^® ^image analysis software.

### DNA isolation and analysis of transgene copy number by real-time PCR

DNA was isolated from transduced K562 cells using QIAmp kit (Qiagen) and concentrations measured with a spectrophotometer. The real time PCR analysis was carried out as previously described using primers specific for sequences located within WPRE region of the vector to determine copy numbers [22]. The average copy number of the transgene in genomic DNA isolated from transduced K562 cells was determined using the ABI 7900 sequence detection system and TaqMan chemistries (Applied Biosystems, Foster City, CA). In all the real time PCR analysis a single-copy eGFP lentiviral transgene containing DNA sample from a clone of 293T cells were included as reference control.

### Statistical analysis

Analysis of variance and Tukey's studentized range test were used to determine the significance of the differences in HIV-1 *Gag *(p24) levels in the vector supernatants.

## Competing interests

The author(s) declare that they have no competing interests.

## Authors' contributions

NC and DC participated in the design of experiments, carried out some of the experiments and supervised the work and wrote the manuscript. MDM and JN constructed the vectors and carried out some of the initial studies. JS produced lentiviral vectors, carried out real time PCR, western and northern blot analysis. AFS contributed on IHC analysis. NC, DC, GPM, MDM and LJF contributed intellectually on the design and interpretation of results and in writing the manuscript.
